# Editorial

**Published:** 1842-10-22

**Authors:** 


### THE MEDICAL EXAMINER.

### PHILADELPHIA, OCTOBER 22, 1842.

### *Fête at Bicêtre.*

A most singular and interesting exhibition recently took place at Bicêtre, the Insane Hospital near Paris. A vaudeville was performed byseveral of the inmates of the establishment, before their comrades. The orchestra was directed by Mr. Florimond Rouger, a young Parisian artist, who has devoted his time and talent to instructing and amusing the insane. A prologue was written and spoken by M. Duclos, a man of letters, completely insane, but whose verses do not betray more incoherence of thought and expression, than those of many other mad poets, whom the public never thought of sending to Bedlam. The vaudeville was "l' Ours et le Pacha." The Pacha was a lypemaniac, who several times during the performance relapsed into his ordinary train of thought, but always resumed his part in turn. Roxelane was played by a young epileptic, who had an attack as she was going on the stage, but which did not cause her to forget a word of her part. The rest of the characters were filled by lunatics and epileptics. The choruses were well sung, and the parts all well played, and, in the language of one who witnessed this unique and thrilling performance, "If you had not been in the secret, you would never have supposed that you were at a play acted by insane, and in the midst of a pit of madmen and epileptics."

